# Hyperactivity and attention deficits in mice with decreased levels of stress-inducible phosphoprotein 1 (STIP1)

**DOI:** 10.1242/dmm.022525

**Published:** 2015-11-01

**Authors:** Flavio H. Beraldo, Anu Thomas, Benjamin Kolisnyk, Pedro H. Hirata, Xavier De Jaeger, Amanda C. Martyn, Jue Fan, Daniela F. Goncalves, Matthew F. Cowan, Talal Masood, Vilma R. Martins, Robert Gros, Vania F. Prado, Marco A. M. Prado

**Affiliations:** 1 Robarts Research Institute, The University of Western Ontario, London, Ontario N6A5B7, Canada; 2 Program in Neuroscience, The University of Western Ontario, London, Ontario N6A5B7, Canada; 3 Department of Molecular and Cell Biology, International Research Center, A.C. Camargo Cancer Center and National Institute for Translational Neuroscience Research Center, Sao Paulo, SP 01508-010, Brazil; 4 Department of Physiology and Pharmacology, The University of Western Ontario, London, Ontario N6A5B7, Canada; 5 Department of Anatomy and Cell Biology, The University of Western Ontario, London, Ontario N6A5B7, Canada

**Keywords:** Touchscreen, Autism, ASD, Stress-inducible phosphoprotein 1, Attention deficits, Mouse model, BAC

## Abstract

Stress-inducible phosphoprotein I (STIP1, STI1 or HOP) is a co-chaperone intermediating Hsp70/Hsp90 exchange of client proteins, but it can also be secreted to trigger prion protein-mediated neuronal signaling. Some mothers of children with autism spectrum disorders (ASD) present antibodies against certain brain proteins, including antibodies against STIP1. Maternal antibodies can cross the fetus blood-brain barrier during pregnancy, suggesting the possibility that they can interfere with STIP1 levels and, presumably, functions. However, it is currently unknown whether abnormal levels of STIP1 have any impact in ASD-related behavior. Here, we used mice with reduced (50%) or increased STIP1 levels (fivefold) to test for potential ASD-like phenotypes. We found that increased STIP1 regulates the abundance of Hsp70 and Hsp90, whereas reduced STIP1 does not affect Hsp70, Hsp90 or the prion protein. Interestingly, BAC transgenic mice presenting fivefold more STIP1 show no major phenotype when examined in a series of behavioral tasks, including locomotor activity, elevated plus maze, Morris water maze and five-choice serial reaction time task (5-CSRTT). In contrast, mice with reduced STIP1 levels are hyperactive and have attentional deficits on the 5-CSRTT, but exhibit normal performance for the other tasks. We conclude that reduced STIP1 levels can contribute to phenotypes related to ASD. However, future experiments are needed to define whether it is decreased chaperone capacity or impaired prion protein signaling that contributes to these phenotypes.

## Abstract (precis)

**Summary:** Here, using a series of behavioral tests including touchscreen tasks we show that decreased levels of stress-inducible phosphoprotein 1 (STIP1) lead to attention deficits and hyperactivity in mice.

## INTRODUCTION

In autism spectrum disorders (ASD), alterations in genetic variance and neurodevelopmental are both thought to contribute to phenotype heterogeneity. Womb environment and autoimmune responses have been proposed to contribute to the complex behavioral alterations observed in ASD, which include, but are not limited to, abnormal socialization and communication and stereotyped behavior (Brimberg et al., 2013; Goldani et al., 2014). Several distinct groups have investigated the existence of antibodies against fetal brain tissue in mothers of ASD children (Bauman et al., 2013; Braunschweig et al., 2012b, 2013; Dalton et al., 2003; Nordahl et al., 2013). Passive transfer of maternal anti-brain antibodies to pregnant experimental animal models (including mice, rats and non-human primates) has shown that their offspring develop a number of endophenotypes that resemble phenotypes in ASD (Bauman et al., 2013; Braunschweig et al., 2012b; Dalton et al., 2003). Indeed, a recent study indicated that the prevalence of antibodies against fetal brain proteins is increased fourfold in mothers of an ASD child compared with control groups (Brimberg et al., 2013). Proteomics analysis has identified six brain proteins as targets for ASD antibodies, including lactate dehydrogenase A and B (LDH), cypin, stress-inducible phosphoprotein protein1 (STIP1), collapsine response mediator proteins 1 and 2 (CRMP1, CRMP2) and Y-box-binding protein (YBX1) (Braunschweig et al., 2013). Interestingly, injection of maternal antibodies that recognize LDH, STIP1 and CRMP1 in developing mouse embryos causes an increase in cortical neural precursor proliferation and cortical neuron volume, with consequent increase in brain size and weight (Martinez-Cerdeno et al., 2014). These phenotypes are consistent with the notion that the presence of maternal autoantibodies can affect neuronal development.

STIP1, also known as heat-shock organizing protein (Hop) or STI1, is a co-chaperone that interacts concomitantly with heat-shock proteins 70 and 90 (Hsp70 and HsP90) (Abbas-Terki et al., 2002; Chen et al., 1996; Nicolet and Craig, 1989; Picard, 2002; Smith et al., 1993). The chaperone machinery is thought to provide a buffer for cells to respond to environmental challenges; disturbance of Hsp70/90 chaperone activity decreases cellular resilience to stress (Chen et al., 2015; Hashimoto-Torii et al., 2014; Taipale et al., 2010, 2014). The absence of STIP1 in mice has important consequences for development, including increased apoptosis, DNA damage and death (Beraldo et al., 2013). These phenotypes are rescued by transgenic BAC expression of STIP1 (Beraldo et al., 2013).

In addition to its intracellular role as a co-chaperone, STIP1 is also secreted by a variety of cells (Erlich et al., 2007; Eustace and Jay, 2004; Hajj et al., 2013; Lima et al., 2007; Wang et al., 2010) via extracellular vesicles (Hajj et al., 2013). Extracellular STIP1 can signal via the prion protein (PrP^C^) to produce a myriad of effects related to brain development (Beraldo et al., 2010, 2013; Caetano et al., 2008; Lopes et al., 2005; Soares et al., 2013). Here, we used *Stip1* heterozygous mice (*STI1*^−/+^ mice), as well as mice overexpresssing four- to fivefold more STIP1 (*STI1*^TGA^ mice), to investigate the consequences of alteration of STIP1 levels *in vivo*. We report that decreased, but not increased, STIP1 levels affect attention and cause hyperactivity in mice, two phenotypes that are related to ASD-like phenotypes. Our results suggest that interference with STIP1 functions, which presumably occur in the presence of STIP1 antibodies, has the potential to contribute to ASD-like phenotypes.

**TRANSLATIONAL IMPACT**

**Clinical issue**

Autism spectrum disorders (ASD) represent a range of neurodevelopmental disorders with no cure. ASD is characterized by difficulties in communication and socialization, repetitive movements, hyperactivity, impulsivity, and an impaired ability to concentrate and attend to simple tasks. Genetic variance and neurodevelopmental alterations are both thought to contribute to the heterogeneity of the ASD phenotype. Recent studies have demonstrated that some mothers of children with ASD produce antibodies against six specific proteins present in the fetal brain; presumably, these antibodies can interfere with protein function in the developing brain. One of these antibodies targets a protein known as stress inducible phosphoprotein 1 (STIP1). Moreover, a polymorphism for STIP1 was recently identified as a potential risk factor in attention deficit hyperactivity disorder, which shares some phenotypes with ASD. STIP1 is a co-chaperone that mediates the Hsp70/Hsp90 exchange of client proteins. It also triggers prion protein-mediated neuronal signaling.

**Results**

Here, to investigate the potential involvement of STIP1 in ASD, the authors examine mice that express reduced (50%) or increased (fivefold) levels of STIP1. They show that increased STIP1 levels regulate the abundance of Hsp70 and Hsp90. By contrast, reduced STIP1 levels have no effect on Hsp70, Hsp90 or prion protein levels. Notably, however, mice expressing increased levels of STIP1 show no major phenotype when examined using a range of behavioral tasks, whereas mice expressing reduced levels of STIP1 exhibit attention deficits and are hyperactive.

**Implications and future directions**

Because attention deficits and hyperactivity are present in ASD, these findings suggest that interference with STIP1 functions (but not increased STIP1 levels) can contribute to ASD-like phenotypes. Changes in STIP1 levels, possibly triggered by the presence of maternal anti-STIP1 antibodies during brain development, might interfere with the development of brain circuits that affect ASD-like behavior. Additional experiments are required to determine whether decreased STIP1 contributes to ASD-like phenotypes by decreasing chaperone capacity in the developing brain, by impairing prion protein signaling, or through some other mechanism, and to define fully the consequences of disturbed STIP1 activity in ASD.

## RESULTS

**Fig. 1.**
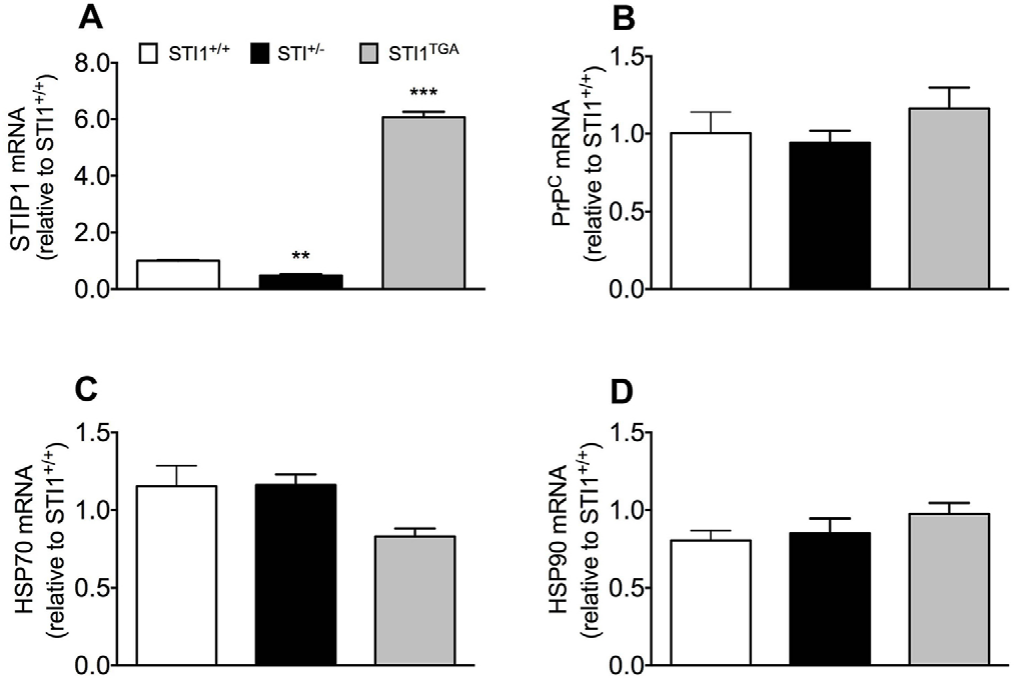
**Analyses of mRNA for STIP1 partners in *STI1*^+/+^, *STI1*^−/+^ and *STI1*^TGA^ mouse brains.** (A) *STIP1* mRNA expression (*n*=9 *STI1*^+/+^, *n*=5 *STI1*^TGA^ and *n*=4 *STI1*^−/+^). (B) *PrP^C^* mRNA expression (*n*=8 *STI1*^+/+^, *n*=4 *STI1*^TGA^ and *n*=7 *STI1*^−/+^). (C) *Hsp70* mRNA expression (*n*=8 *STI1*^+/+^, *n*=4 *STI1*^TGA^ and *n*=7 *STI1*^−/+^). (D) *Hsp90* mRNA expression (*n*=3 *STI1*^+/+^, *n*=4 *STI1*^TGA^ and *n*=4 *STI1*^−/+^). Results are presented as means±s.e.m.; data were analyzed and compared by one-way ANOVA and Bonferroni multiple comparisons post-hoc test; **P<0.001 and ***P<0.0001 compared with control.

We initially confirmed previous data to show that *STI1*^−/+^ mice present 50% of *STIP1* mRNA levels in their brain, whereas *STI1*^TGA^ mice express almost sixfold more mRNA (Fig. 1A; one-way ANOVA; revealed main effect of genotype *F*_(2.15)_=8.521, *P*<0.0001). In contrast, mRNA levels of known STIP1 interaction partners PrP^C^ (Fig. 1B; one-way ANOVA *F*_(2.16)_=1.475, *P*=0.2580), Hsp70 (Fig. 1C; one-way ANOVA *F*_(2.16)_=0.301, *P*=0.744) and Hsp90 (Fig. 1D; one-way ANOVA *F*_(2.8)_=1.249, *P*=0.337) were not altered in the brain of the two lines, compared with control mice.

**Fig. 2.**
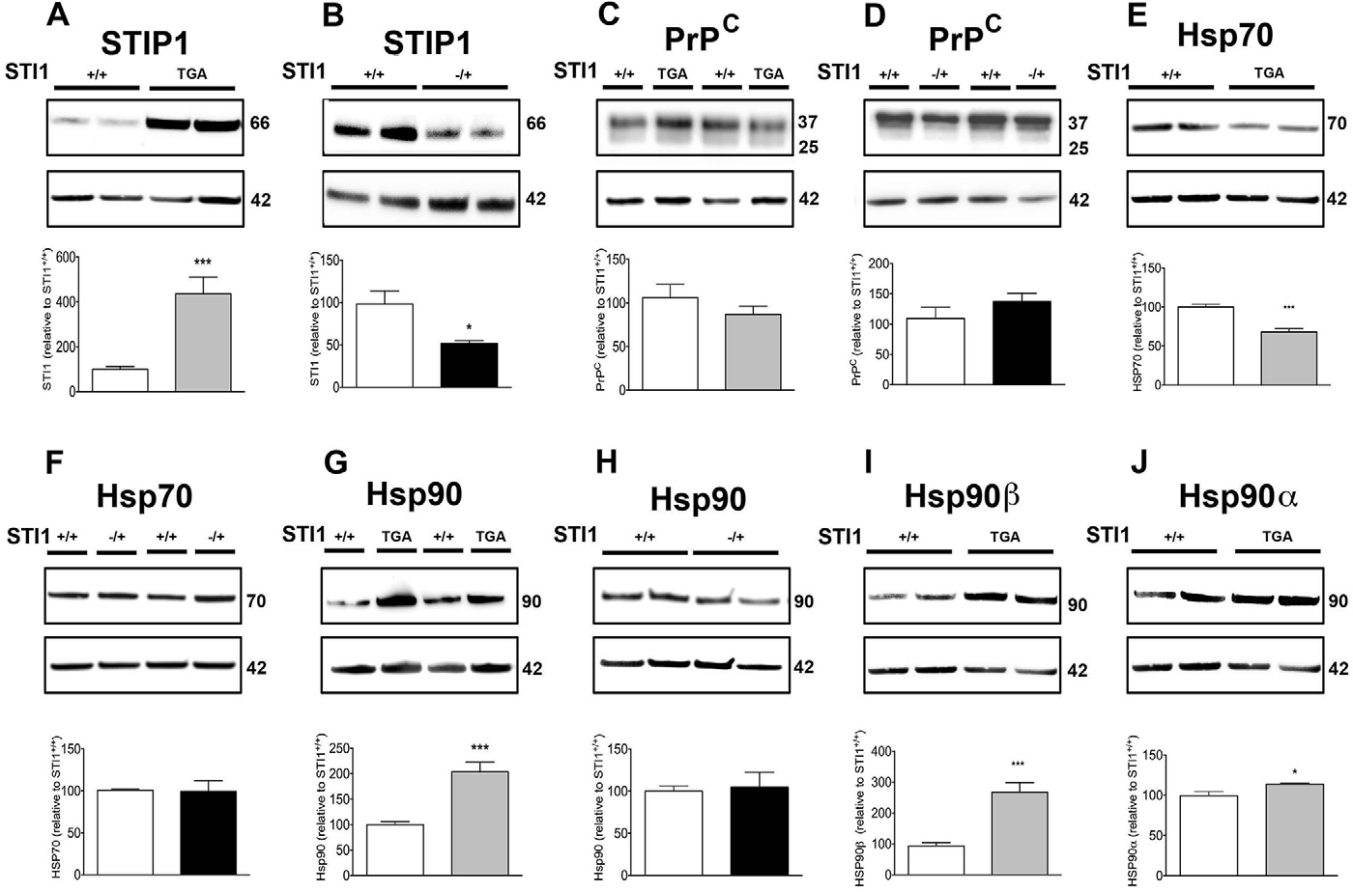
**Analyses of protein levels for STIP1 partners in *STI1*^+/+^, *STI*^−/+^ and *STI1*^TGA^ mouse brains.** (A,B) STIP1 expression in *STI1*^TGA^ (*n*=9 *STI1*^+/+^ and *n*=8 *STI1*^TGA^) and *STI1*^−/+^ mice (*n*=8 *STI1*^+/+^ and *n*=8 *STI1*^−/+^). (C,D) PrP^C^ expression in *STI1*^TGA^ (*n*=6 *STI1*^+/+^ and *n*=6 *STI1*^TGA^) and *STI1*^−/+^ mice (*n*=6 *STI1*^+/+^ and *n*=9 *STI1*^−/+^). (E,F) Hsp70 expression in *STI1*^TGA^ (*n*=5 *STI1*^+/+^ and *n*=4 *STI1*^TGA^) and *STI1*^−/+^ mice (*n*=5 *STI1*^+/+^ and *n*=4 *STI1*^−/+^). (G,H) HSP90 expression in *STI1*^TGA^ (*n*=10 *STI1*^+/+^ and *n*=14 *STI1*^TGA^) and *STI1*^−/+^ mice (*n*=6 *STI1*^+/+^ and *n*=6 *STI1*^−/+^). (I,J) Hsp90β (*n*=10 *STI1*^+/+^ and *n*=8 *STI1*^TGA^) and Hsp90α (*n*=5 *STI1*^+/+^ and *n*=4 *STI1*^TGA^) in *STI1*^TGA^ mice. Results are presented as means±s.e.m.; data were analyzed and compared by Student's *t-*test; *P<0.05 and ***P<0.0001 compared with control.

Protein levels for STIP1 followed mRNA levels for both *STI1*^TGA^ (Fig. 2A; t_(15)_=4.721, *P*=0.003) and *STI1*^−/+^ (Fig. 2B; t_(14)_=6.433, *P*<0.0001). PrP^C^ protein levels were not different from controls in both lines (Fig. 2C,D; t_(10)_=1.049, *P*=0.391 and t_(13)_=1.128, *P*=0.279, respectively). Interestingly, levels of Hsp70 were decreased by 50% in *STI1*^TGA^ brains (Fig. 2E; t_(7)_=5.846, *P*=0.0006), whereas no change in Hsp70 levels was detected in *STI1*^−/+^ mice (Fig. 2F; t_(7)_=0.123, *P*=0.9051), compared with controls. Additionally, Hsp90 levels detected with a pan Hsp90 antibody were doubled in *STI1*^TGA^ brains (Fig. 2G; t_(22)_=4.618, *P*=0.0001) but not changed in *STI1*^−/+^ brains (Fig. 2H; t_(10)_=0.308, *P*=0.7639), compared with controls. We then evaluated expression levels of Hsp90α (inducible form) and Hsp90β (constitutive form) in the brains of *STI1*^TGA^ mice and observed that both forms were significantly increased (Fig. 2I,J; t_(22)_=4.618, *P*=0.0016 and t_(16)_=5.954, *P*<0.0001, respectively).

**Fig. 3.**
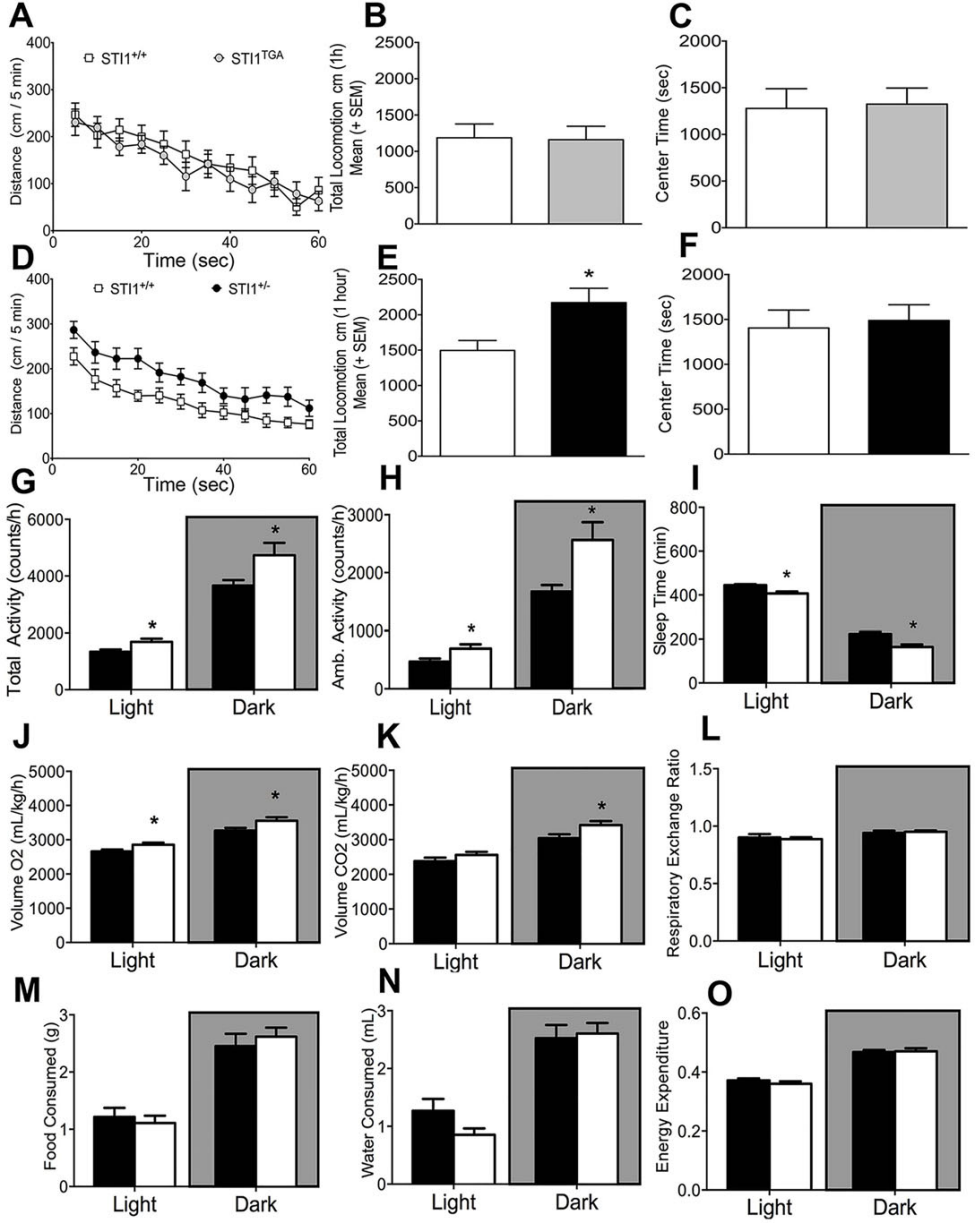
**Locomotor activity in *STI1*^TGA^ and *STI1*^−/+^mice and metabolic analyses in *STI1*^−/+^mice.** (A) Horizontal locomotor activity in an open-field for *STI1*^TGA^ (*n*=14) and *STI*^+/+^ control mice (*n*=14). (B) Cumulative 1 h locomotion for *STI1*^TGA^ (*n*=14) and *STI*^+/+^ control mice (*n*=14). (C) Time spent in the center of the locomotion boxes for *STI1*^TGA^ (*n*=14) and *STI*^+/+^ control mice (*n*=14). (D) Horizontal locomotor activity in an open-field for *STI1*^−/+^ (*n*=8) and *STI*^+/+^ control mice (*n*=8). (E) Cumulative 1 h locomotion for *STI1*^−/+^ (*n*=22) and *STI*^+/+^ control mice (*n*=24). (F) Time spent in the center of the locomotion boxes for *STI1*^−/+^ (*n*=22) and *STI*^+/+^ control mice (*n*=24). (G) Total activity in metabolic cages for *STI1*^−/+^ (*n*=8) and *STI*^+/+^ control mice (*n*=8). (H) Ambulatory activity in metabolic cages for *STI1*^−/+^ (*n*=8) and *STI*^+/+^ control mice (*n*=8). (I) Sleep time for *STI1*^−/+^ (*n*=8) and *STI*^+/+^ control mice (*n*=8). (J) VO_2_ for *STI1*^−/+^ (*n*=8) and *STI*^+/+^ control mice (*n*=8). (K) VCO_2_ for *STI1*^−/+^ (*n*=8) and *STI*^+/+^ control mice (*n*=8). (L) Respiratory exchange ratio for *STI1*^−/+^ (*n*=8) and *STI*^+/+^ control mice (*n*=8). (M) Food consumption for *STI1*^−/+^ (*n*=8) and *STI*^+/+^ control mice (*n*=8). (N) Water consumption for *STI1*^−/+^ (*n*=8) and *STI*^+/+^ control mice (*n*=8). (O) Energy expenditure for *STI1*^−/+^ (*n*=8) and *STI*^+/+^ control mice (*n*=8). Results are presented as means±s.e.m.; data were analyzed and compared by Student's *t-*test; *P<0.05 compared with control.

Spontaneous locomotor activity in a new environment can provide information on neuropsychiatric phenotypes in mice associated with genetic mutations. The increased number of *Stip1* copies, with concomitant overexpression of Hsp90 and decreased expression of Hsp70 in *STI1*^TGA^ mice did not seem to have any major impact on spontaneous locomotion (Fig. 3A,B; t_(29)_=1.140, *P*=0.942) or time spent in the center of the box, which provides insight on anxiety-like behavior (Fig. 3C; t_(29)_=1.236, *P*=0.8669). In contrast, locomotor activity and total locomotion in a new environment were increased in *STI1*^−/+^ mice (Fig. 3D,E; t_(44)_=1.879, *P*=0.0078). However, *STI1*^−/+^ mice did not show increased anxiety-like behavior, as determined by the time spent in the center of the box (Fig. 3F; *t*_(40)_=1.221, *P=*0.341). We also examined another cohort of *STI1*^−/+^ mice using automated metabolic cages. In this experiment, which mimics the home cage environment, *STI1*^−/+^ mice again showed hyperactivity during the day and night periods, considering both total activity (Fig. 3G; *t*_(14)_*=*2.558, *P*=0.0228 and *t*_(14)_*=*2.230, *P*=0.0426) and ambulatory activity (Fig. 3H; *t*_(14)_*=*2.420, *P*=0.00297 and *t*_(14)_*=*2.230, *P*=0.0426). Given this increased motor activity, *STI1*^−/+^ mice also demonstrated less sleep time (periods of inactivity) (Fig. 3I; *t*_(14)_=3949, *P*=0.0015 and *t*_(14)_=2.724, *P*=0.0165). Also, *STI1*^−/+^ mice showed increased consumption of O_2_ during the light and dark cycle (Fig. 3J; *t*_(14)_=2.464, *P*=0.027 and *t*_(14)_=2.169, *P*=0.047) and CO_2_ production during the dark cycle, but not in the light cycle (Fig. 3K; *t*_(14)_=2.307, *P*=0.036 and *t*_(14)_=1.360, *P*=0.195). No differences were observed in other parameters such as respiratory ratio (Fig. 3L; *t*_(14)_=0.4455, *P*=0.6627 and *t*_(14)_=0.459, *P*=0.653), food consumption (Fig. 3M; *t*_(14)_=0.5216, *P*=0.6101 and *t*_(14)_=0.6134, *P*=0.5494), water consumption (Fig. 3N; *t*_(14)_=1.801, *P*=0.0933 and *t*_(14)_=0.2752, *P*=0.7872), and heat production (Fig. 3O; *t*_(14)_=1.014, *P*=0.3276 and *t*_(14)_=0.1935, *P*=0.8494) comparing *STI1*^−/+^ to *STI1*^+/+^ mice for both cycles (light and dark).

**Fig. 4.**
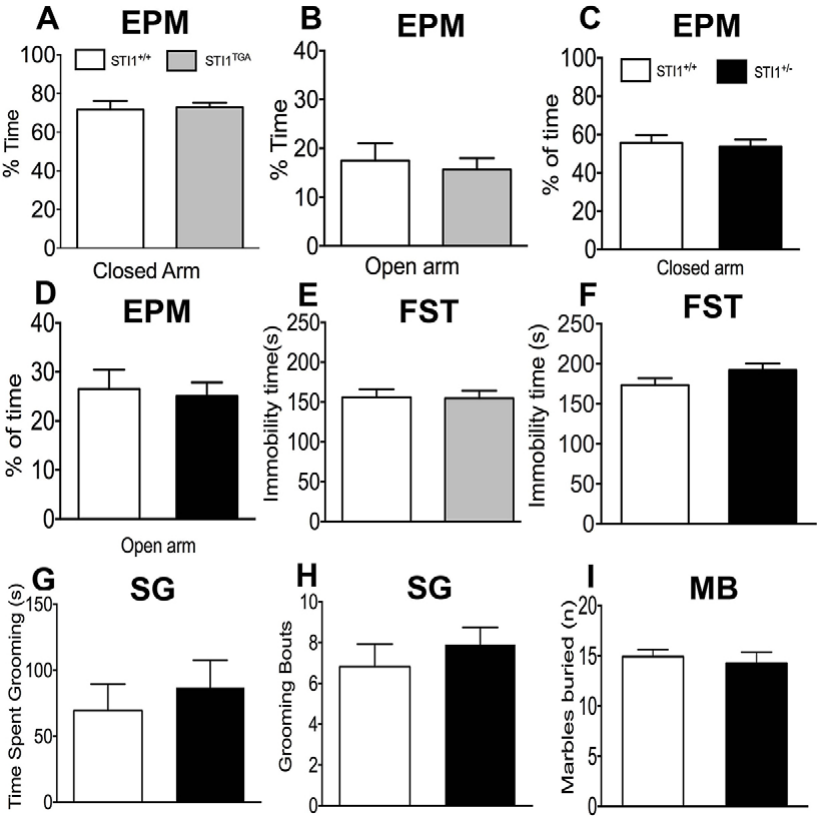
**Anxiety-like behavior, depression-like behavior, social behavior and compulsivity in *STI1*^TGA^ and *STI1*^−/+^ mice.** (A) Percentage of time spent in the closed arm for *STI1*^TGA^ (*n*=17) and control mice (*n*=14). (B) Percentage of time spent in the open arm for *STI1*^TGA^ (*n*=17) and control mice (*n*=14). (C) Percentage of time spent in the closed arm for *STI1*^−/+^ (*n*=13) and control mice (*n*=10). (D) Percentage of time spent in the open arm for *STI1*^−/+^ (*n*=13) and control mice (*n*=10). (E) Immobility time in the forced-swimming test for *STI1*^TGA^ (*n*=17) and control mice (*n*=14). (F) Immobility time in the forced-swimming test for *STI1*^−/+^ (*n*=6) and control mice (*n*=8). (G,H) Time spent grooming and number of grooming bouts for *STI1*^−/+^ (*n*=11) and control mice (*n*=11). (I) Marbles buried by *STI1*^−/+^ (*n*=12) and control mice (*n*=12).

In order to test for other neuropsychiatric-like behaviors as a result of altered STIP1 levels we tested both *STI*^TGA^ and *STI1*^−/+^ mice for anxiety-like behavior (Fig. 4A-D) and depression-like behavior (Fig. 4E,F). Given the hyperactivity of *STI1*^−/+^ mice, we also decided to investigate whether they had alterations in compulsive-like behavior, assessed by measurement of self-grooming and marble burying (Fig. 4G-I). There was no difference in the behavior of either *STI*^TGA^ (Fig. 4A,B,E) or *STI1*^−/+^ (Fig. 4C,D,F-I) mice compared with control mice in all these behavioral tasks: time spent in the open arm (Fig. 4C; t_19_=0.310, *P*=0.7590), time spent in the closed arm (Fig. 4D; t_(19)_=0.3730, *P*=0.7133), forced swim test (Fig. 4F; t_(12)_=1.184, *P*=0.2594), grooming bouts (Fig. 4H; t_(20)_=0.7848, *P*=0.4418), time grooming (Fig. 4G; t_(20)_=0.6072, *P*=0.5505) and marble burying (Fig. 4I; t_(21)_=0.4956, *P*=0.6253).

**Fig. 5.**
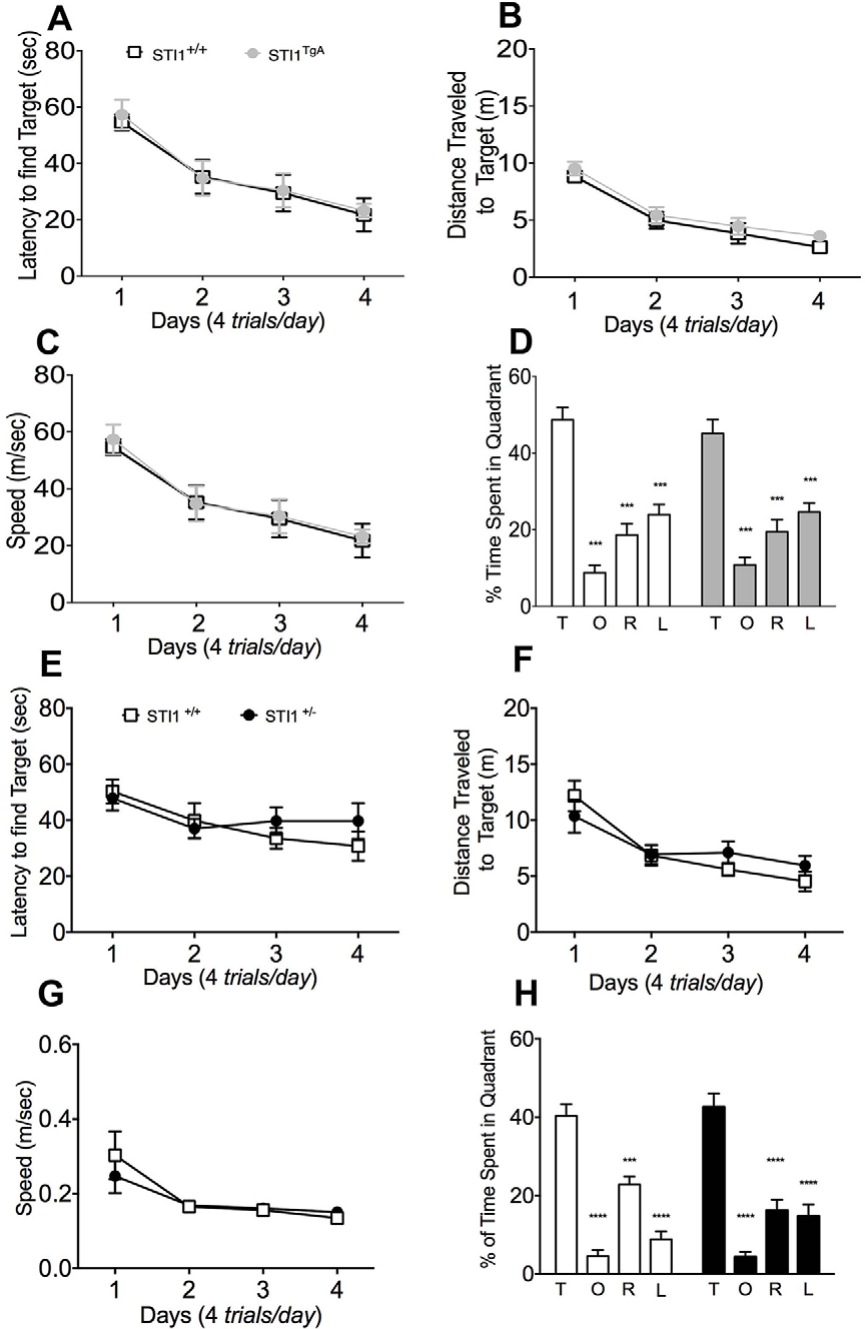
**Spatial memory in *STI1*^TGA^ and *STI1*^−/+^ mice.** For the tests, *n*=14 *STI1*^+/+^ and 14 *STI1*^TGA^ mice were used to test spatial memory in *STI1*^TGA^ mice and *n*=11 *STI1*^+/+^ and 11 *STI1*^−/+^ for *STI1*^−/+^ mice. (A) Latency to find the platform. (B) Distance traveled. (C) Speed for *STI1*^TGA^ mice. (D) Percentage time spent by *STI1*^TGA^ mice and controls in target quadrant (T) and in opposite (O), right (R) and left (L) quadrants was measured on day 5 in a 60 s probe trial with the platform removed. (E) Latency to find the platform. (F) Distance traveled. (G) Speed for *STI1*^−/+^ mice. (H) Percentage time spent by *STI1*^−/+^ mice and controls in each quadrant was measured on day 5 in a 60 s probe trial with the platform removed. Results are presented as means±s.e.m.; data were analyzed and compared by two-way ANOVA; ***P<0.001 and ****P<0.0001 compared with time spent in target quadrant.

Next, we investigated spatial navigation memory in both *Stip1* mutant mice using the Morris water maze (MWM). Neither *STI1*^TGA^ nor *STI1*^−/+^ mice presented deficits in acquisition or retrieval of spatial memory in the MWM. For both *STI1*^TGA^ and *STI1*^−/+^, performance during the 4-day acquisition phase was indistinguishable from their wild-type controls in terms of latency to find the target (Fig. 5A; RM-ANOVA *F*_(1,13)_=0.062, *P*=0.806) or speed (Fig. 5C; *F*_(1,10)_=0.215, *P*=0.652). When spatial memory retrieval was performed on the day-5 probe trial, again no differences were observed between *STI1*^TGA^ and *STI1*^−/+^ mice, compared with their wild-type controls, for time spent investigating the target quadrant (Fig. 5D; *F*_(1,13)_=1.046, *P*=0.3251) or latency (Fig. 5E; *F*_(1,10)_=0.215, *P*=0.294).

**Fig. 6.**
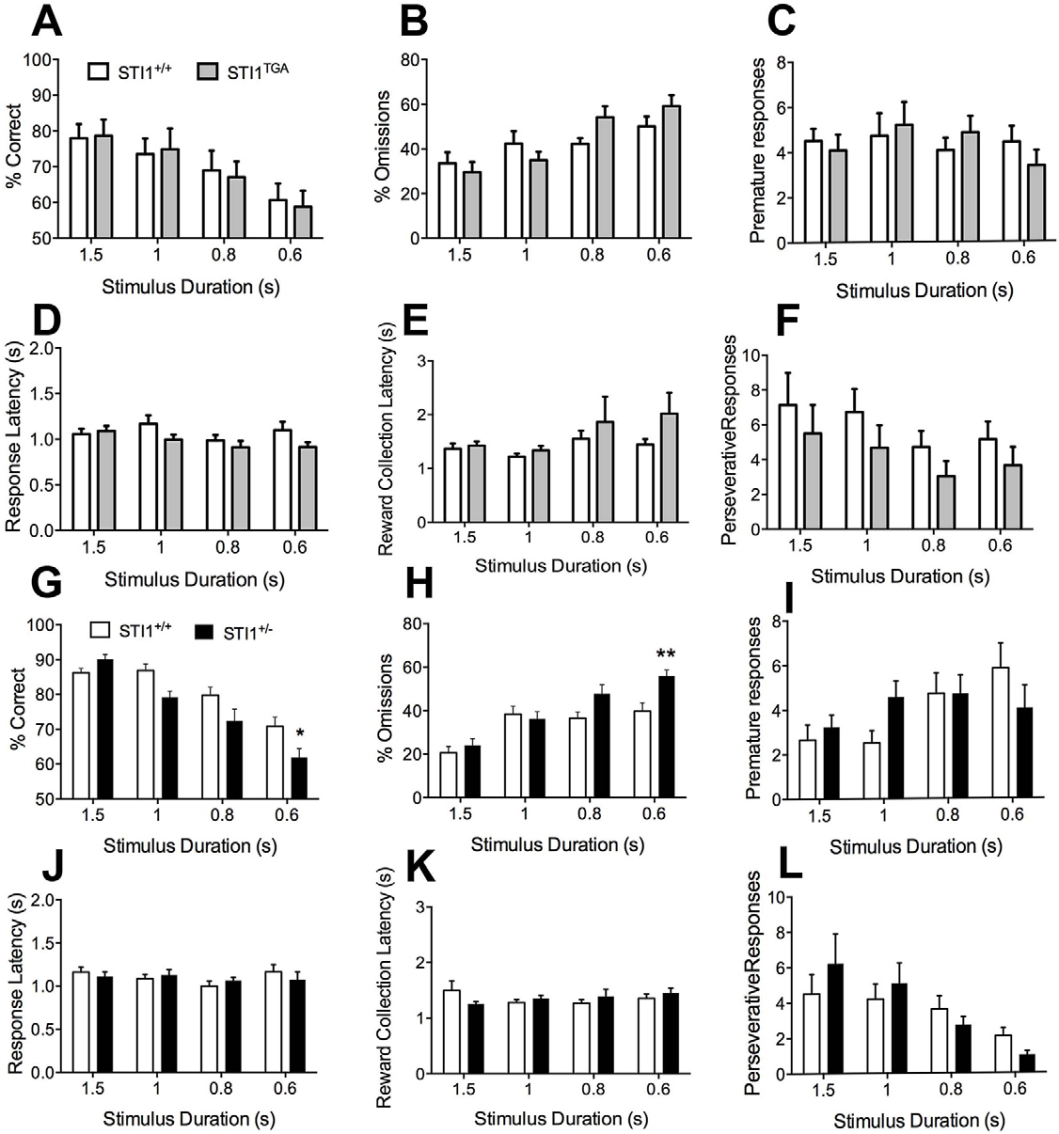
**Five-choice serial reaction time task used to measure attention in *STI1*^TGA^ and *STI1*^−/+^.** For the tests, *n*=10 *STI1*^+/+^ and 10 *STI1*^TGA^ mice were used to test attention in *STI1*^TGA^ mice and *n*=13 *STI1*^+/+^ and 13 *STI1*^−/+^ for *STI1*^−/+^ mice. (A) Accuracy during probe trial sessions. (B) Rate of omission. (C) Premature responses. (D) Response latency. (E) Reward collection latency. (F) Perseverative responses for *STI1*^TGA^ mice. (G) Accuracy during probe trial sessions. (H) Rate of omission. (I) Premature responses. (J) Response latency. (K) Reward collection latency. (L) Perseverative response for *STI1*^−/+^ mice. Results are presented as means±s.e.m.; data were analyzed and compared by RM-ANOVA; *P<0.05, **P<0.001 compared with control.

Given the hyperactivity phenotype and genetic data suggesting the potential of STIP1 to be linked to ADHD (Mick et al., 2011), a trait commonly found in ASD (Gadow et al., 2006; Goldstein and Schwebach, 2004; Lee and Ousley, 2006; Mulligan et al., 2009), we also determined whether changes in STIP1 levels affected attentional processing. For this, we used the 5-CSRTT. After mice were trained to perform to a criterion (>80% accuracy, <20% omissions) at a 2 s stimulus duration, we assessed attentional performance by using reduced stimulus durations in probe trials (1.5, 1, 0.8 and 0.6 s stimulus durations) as previously described (Romberg et al., 2011). We observed no differences in attentional performance in *STI1*^TGA^ mice compared with their littermate controls. There was no difference in accuracy (Fig. 6A; RM-ANOVA showed no effect of genotype *F*_(1,20)_=0.0057, *P*=0.9403, main effect of stimulus duration *F*_(3,60)_=12.14, *P*<0.0001 and no significant interaction *F*_(3,60)_=0.1328, *P*=0.9402) or omission rates (Fig. 6B; RM-ANOVA showed no effect of genotype *F*_(1,18)_=0.2429, *P*=0.6281, main effect of stimulus duration *F*_(3,54)_=17.62, *P*<0.0001 and significant interaction *F*_(3,54)_=3.854, *P*=0.0143). Post-hoc analysis showed that there was no significant difference between *STI1*^TGA^ mice and controls. There was also no difference in premature responses, a measure of impulsivity (Fig. 6C; RM-ANOVA showed no effect of genotype *F*_(1,9)_=0.00056, *P*=0.9419, no effect of stimulus duration *F*_(3.27)_=0.8254, *P*=0.4914 and no significant interaction *F*_(3,27)_=1.109, *P*=0.3625). Moreover, we did not find any difference in motivation, measured as latency to touch the screen (Fig. 6D; RM-ANOVA showed no effect of genotype *F*_(1,9)_=3.399, *P*=0.0983, main effect of stimulus duration *F*_(3.30)_=4.281, *P*=0.0125 and no significant interaction *F*_(3,30)_=2.332, *P*=0.0941). Compulsivity and motivation were not altered either, as assessed by perseverative responses (Fig. 6F; RM-ANOVA, showed no effect of genotype *F*_(1,9)_=3.974, *P*=0.0774, main effect of stimulus duration *F*_(3,27)_=4.808, *P*=0.0083 and no significant interaction *F*_(3,27)_=0.1773, *P*=0.9108) and reward collection latency (Fig. 6E; RM-ANOVA showed no effect of genotype *F*_(1,10)_=1.291, *P*=0.2824, no effect of stimulus duration *F*_(3,30)_=2.162, *P*=0.1132 and no significant interaction *F*_(3.30)_=0.7372, *P*=0.5381).

In contrast, when attentional demand was increased, *STI1*^−/+^ mice presented decreased accuracy (Fig. 6G; RM-ANOVA, main effect of genotype *F*_(1,25)_=6.872, *P*=0.0147, main effect of stimulus duration *F*_(3,75)_=41.95, *P*<0.0001, significant interaction effect *F*_(3,75)_=4.170, *P*=0.0087) and increased omission rates (Fig. 6H; RM-ANOVA, main effect of genotype *F*_(1,25)_=6.584, *P*=0.0167, main effect of stimulus duration *F*_(3,75)_=24.62, *P*<0.0001, significant interaction effect *F*_(3,75)_=3.401, *P*=0.0220). Post-hoc analysis revealed that the *STI1*^−/+^ mice were significantly impaired in both accuracy and omissions at the 0.6 s stimulus duration. The worse performance of *STI1*^−/+^ mice was not related to changes in motivation (latency to respond to the stimulus, RM-ANOVA, no effect of genotype *F*_(1,25)_=0.01856, *P*=0.8925, no effect of stimulus duration *F*_(3,75)_=1.720, *P*=0.1702, no interaction *F*_(3,75)_=1.070, *P*=0.3669). There was also no difference in latency to retrieve the reward following a correct response (RM-ANOVA, no effect of genotype *F*_(1,25)_=0.03176, *P*=0.8600, no effect of stimulus duration *F*_(3,75)_=0.3997, *P*=0.7536, no interaction *F*_(3,75)_=1.785, *P*=0.8284). Moreover, we detected no increase in premature responses (RM-ANOVA, no effect of genotype *F*_(1,25)_=0.0958, *P*=0.7595, main effect of stimulus duration *F*_(3,75)_=2.907, *P*=0.0401, no interaction effect *F*_(3,75)_=2.017, *P*=0.1187) or perseverative responses (RM-ANOVA, no effect of genotype *F*_(1,25)_=0.04188, *P*=0.8395, main effect of stimulus duration *F*_(3,75)_=6.975, *P*=0.0003, no interaction effect *F*_(3,75)_=1.139, *P*=0.3389).

## DISCUSSION

The present experiments tested whether alterations in STIP1 levels have consequences for psychiatric-like behaviors in mice. Our results suggest that decreased, but not increased, STIP1 levels cause significant behavioral alterations in mice. Spatial learning and memory, as well as anxiety and depression-like behavior do not seem to be affected by reduced STIP1 levels. However, mutant mice deficient for STIP1 are hyperactive and present attention deficits.

STIP1 has recently emerged as a protein of potential interest in ASD and endophenotypes related to ASD. Maternal autoantibodies against STIP1 have been identified in mothers of children with ASD (Braunschweig et al., 2013). Moreover, recent global-wide association study (GWAS) analysis identified a polymorphism in *STIP1* (the human gene coding for STIP1/HOP) as a potential risk factor in a population of individuals diagnosed with attention-deficit disorder (Mick et al., 2011), a co-morbidity often associated with ASD (Brimberg et al., 2013; Goldani et al., 2014). The consequences of this polymorphism for STIP1 expression is unknown, but the presence of autoantibodies against STIP1 might affect expression levels of the protein, given that antibodies can penetrate the blood brain barrier of the fetus during pregnancy (Braunschweig et al., 2012a; Diamond et al., 2009; Fox et al., 2012; Zhang et al., 2012). Indeed, maternal antibodies that recognize STIP1 and other targets when injected in pregnant rodents or developing pups can lead to offspring with abnormal neurons and behaviors that relate to ASD (Braunschweig et al., 2012b; Camacho et al., 2014). To a degree, *STI1*^−/+^ mice model this early developmental deficit in STIP1 levels. However, in *STI1*^−/+^ mice STIP1 expression is persistently decreased through life, which could also have important consequences for the phenotypes described.

STIP1 is a modular protein containing several tetratricopeptide (TRP) repeat domains and aspartate-proline (DP) reach domains (Taipale et al., 2010). TRP1 and TRP2B can interact with Hsp70 (Flom et al., 2007; Scheufler et al., 2000), whereas TPR2A is required for interaction with Hsp90 (Flom et al., 2007, 2006). Hsp90 activity is regulated by STIP1 and previous work has shown that in mice no other co-chaperone can replace STIP1 (Beraldo et al., 2013). Recent experiments have indicated that the chaperone machinery, activated by the transcription factor heat shock factor 1 (HSF1), is responsible for preventing damaging effects from environmental factors in the developing brain (Hashimoto-Torii et al., 2014). Indeed, the chaperone machinery can buffer many stresses at the cellular level and, therefore, it is not surprising that functional changes in its components have physiological consequences.

In addition to its intracellular chaperone function, STIP1 is also secreted by a myriad of cells, including astrocytes via an extracellular vesicle population, which includes exosomes (Hajj et al., 2013). Extracellular STIP1 also mediates important physiological responses in the brain. Acting as a trophic factor to engage PrP^C^ to signal in neurons, it regulates neuritogenesis and neuronal survival (Beraldo et al., 2010; Lopes et al., 2005; Roffe et al., 2010). STIP1 has a role in functional recovery in stroke (Beraldo et al., 2013; Lee et al., 2013). Moreover, STIP1 also modulates toxicity of Aβ peptides in models of Alzheimer's disease (Brehme et al., 2014; Ostapchenko et al., 2013).

It is remarkable that mice with increased levels of STIP1 (up to almost fivefold) do not present any major behavioral alteration. In the extensive evaluation of cognitive phenotypes in this study, which included anxiety and depression-like behaviors, spatial memory and attention, we showed that *STI1*^TGA^ mice perform as well as littermate controls. These results suggest that strategies to increase STIP1 levels should not cause toxicity with consequences for brain functions. This is important, given that increased STIP1 levels might be protective against insults such as stroke-mediated cell death and in Alzheimer's disease (Beraldo et al., 2013; Ostapchenko et al., 2013). Interestingly, whereas increased levels of STIP1 seem to affect the chaperone machinery, prion protein expression is not affected by decreasing the level of Hsp70 and increasing Hsp90. These consequences of increased STIP1 seem to occur at the post-translational level, given that mRNAs for Hsp70 and 90 were not affected. It is unknown at the moment whether increased STIP1 levels stabilize a complex containing Hsp90, preferentially leading to increased turnover of Hsp70.

At present, the exact mechanism by which decreased STIP1 levels affect psychiatric-like behavior is still unknown. Although it is possible that decreased levels of STIP1 during early development have persistent effects in brain circuits, culminating with hyperactivity and attentional deficits, we cannot discard the possibility that STIP1 plays a role in regulating circuitry function in the adult brain. Our experiments at the moment do not discriminate whether the phenotypes observed in mutant mice result from decreased STIP1co-chaperone function, diminished STIP1 extracellular signaling or both. Our results suggest that reduced levels of STIP1 have important consequences for behavior and seem to affect brain circuits that regulate attention. It is possible that exposure to STIP1 antibodies during pregnancy could reduce STIP1 levels, which, based on the present results, would have important consequences. Future experiments are required to define potential mechanisms as well as the consequences of disturbed STIP1 activity in ASD.

## MATERIALS AND METHODS

### Animals

*STI1*^−/+^ and *STI1*^TGA^ mice were generated as described (Beraldo et al., 2013). Both mouse lines were in the C57BL/6J background. All experimental procedures were conducted in compliance with the Canadian Council of Animal Care guidelines for use and care of animals and in accordance with approved animal use protocols at the University of Western Ontario (2008/127). Animals were housed in groups of two or four per cage. Mice were kept in a temperature-controlled room with a 12/12 light/dark cycle (7 am/7 pm) with food and water provided *ad libitum* unless stated otherwise. For behavioral studies, only male mice were used. Mice were randomized and the experimenter was blind to genotypes. For most of the behavioral tasks, software-based analyses were used to score mice performance with minimum human interference.

### qPCR and Western blot

For real-time quantitative PCR (qPCR), brain tissues were homogenized in Trizol and total RNA was extracted using the Aurum Total RNA kit for fatty and fibrous tissue (Bio-Rad, Hercules, CA, USA). qPCR were performed as previously described (Martins-Silva et al., 2011). Primer sequences: STIP1-F, 5′-GCCAAGAAAGGAGACTACCAG-3′; STIP1-R, 5′-TCATAGGTTCGTTTGGCTTCC-3′; HsP90-F, 5′-CCACCCTGCTCTGTACTACT-3′; HsP90-R, 5′-CCAGGGCATCTGAAGCATTA-3′; HsP70-R, 5′-ACCTTGACAGTAATCGGTGC-3′; HsP70-F, 5′-CTCCCGGTGTGGTCTAGAAA-3′; PRP-F, 5′-GAACCATTTCAACCGAGCTG-3′; PRP-R, 5′-CATAGTCACAAAGAGGGCCAG-3′; Actin-F, 5′-TGGAATCCTGTGGCATCCATGA-3′; and Actin-R, 5′-AATGCCTGGGTACATGGTGGTA-3′. Immunoblot analysis was carried out as described previously (Beraldo et al., 2013). The antibodies used were anti-STIP1 (1:5000, in-house antibody generated by Bethyl Laboratories Montgomery, USA using recombinant STIP1) (Beraldo et al., 2013), anti-Hsp90 (1:1000), anti-Hsp70 (1:1000), anti-Hsp90α (1:1000), anti Hsp90β (1:1000) (Cell Signaling, Danvers, USA) and anti-PrP 8H4 (1:2000) (Abcam, Cambrige, UK).

### Locomotor activity

Mice were acclimated to the testing room for 30 min prior to beginning the test; locomotor activity was automatically recorded (Omnitech Electronics Inc., Columbus, USA). Mice were placed in the center of the apparatus and locomotor activity was measured at 5 min intervals for 1 h as described previously (Martyn et al., 2012).

### Elevated plus maze

To access anxiety-like behavior, mice were acclimated to the testing room for 30 min prior to beginning the test and then placed in the center of the elevated plus maze (Med Associates Inc., St Albans, USA). The activity was recorded and videos were analyzed using ANY-maze software (Stoelting Co., USA) to determine the amount of time spent in the closed and open sections of the maze.

### Forced swimming test

Depressive-like behavior was assessed by a forced swim test (FST) as described previously (Martyn et al., 2012). Briefly, mice were placed in a 2 l beaker containing 1.7 l of water at 25-27°C for 6 min. Experimental sessions were recorded and immobility time was evaluated using ANY-Maze Software (Stoelting Co., USA). Data obtained from the last 4 min of testing were used for the analysis.

### Morris water maze

The spatial version of Morris water maze (MWM) was conducted as described previously (Kolisnyk et al., 2013; Martyn et al., 2012; Vorhees and Williams, 2006). Briefly, the task was performed in a 1.5-m diameter/1-m deep pool filled with water at 25°C. Spatial cues, 40×40 cm boards containing black symbols (vertical and horizontal stripes, triangles, squares and circles), were placed on the walls distributed around the pool and the platform was submerged 1 cm below the surface of the water. Mice were submitted to four training trials a day (90 s each) for four consecutive days with a 15 min intertrial interval. On day 5, memory was assessed by a single 60 s trial on which the platform was removed and the time spent in the target quadrant was evaluated. All the experimental sessions were recorded and analyzed using the ANY-Maze Software.

### Five-choice serial reaction time task

The five-choice serial reaction time task (5-CSRTT) was used to evaluate attention in mice as described previously (Kolisnyk et al., 2013; Romberg et al., 2011). Mice were trained in the 5-CSRTT in automated Bussey–SaksidaTouch screen systems (Campden Instruments Limited, Loughborough, EN) and the data collected using ABET II Touch software V.2.18 (Lafayette Instruments, Lafayette, USA). Mice were submitted to a pre-training program, which consisted of first habituating the mouse to the testing chamber with the lights off for 10 min. The next day, the mouse was put in the chamber with the lights off for 20 min. After two days of habituation with no reward been offered, the reward tray was primed with 11% fat strawberry milkshake (Nielson - Saputo Dairy Products) and a tone was played when the mouse entered the reward tray. This was repeated for the next 2 days for 40 min sessions. Whenever the mouse returned to the reward tray, the reward was offered and paired with a tone (phase I). The following training phase consisted in pairing the reward with the presentation of a random stimulus (flash of light in one of the five windows), which is removed after 30 s. At this phase, if the mouse touched the screen when the stimulus was displayed, it received a reward. This cycle was repeated until the mouse completed 30 trials or 60 min timeout (phase II). At phase III of the training, the stimulus was displayed randomly in one of the five windows. The mouse had to touch the window where the stimulus was displayed to receive the reward paired with a tone. Similar to phase II, this cycle was repeated until the mouse completed 30 trials or 60 min timeout. The next step (phase IV) was identical to phase III except by the fact that the mouse had to poke its nose into the reward trail to initiate the task. This process was repeated in the last phase of the pre-training (phase V); however, if the mouse touched an incorrect screen, it received a 5 s timeout and the light in the chamber was turned on. After the mouse had finished pre-training and reached criterion at 4 s and 2 s stimulus duration (80% accuracy, 20% omission for three consecutive days), mice were probed for attention deficits following probe trial schedules: each mouse was tested over two sessions at 1.5, 1.0, 0.8 and 0.6 s stimulus duration (the order of the probe trial sessions was randomized and the groups counterbalanced). Between each different stimulus duration, each mouse was returned to a 2 s stimulus for two consecutive sessions. Number of trials to criterion, accuracy, omission, reward collection latency and perseverative response were analyzed.

### Metabolic assessments

Oxygen consumption, carbon dioxide production, respiratory exchange ratio (RER), carbon dioxide production, water and food intake and physical activity were simultaneously measured for adult *STI1*^+/+^ and *STI1*^+/−^ mice by using the Comprehensive Lab Animal Monitoring System (CLAMS) interfaced with Oxymax Software (Columbus Instruments, Columbus, OH, USA) as previously described in detail (Guzman et al., 2013; Kolisnyk et al., 2013). Briefly, mice were individually housed in the metabolic chambers with *ad libitum* access to water and food. Following a 16-h habituation period, all measurements were obtained every 10 min for 24 h (12 h light/12 h dark).

### Marble burying task

A marble burying task was used to assess repetitive and anxiety-like behavior as previously described (Deacon, 2006).

### Assessment of self-grooming

Self-grooming was assessed to evaluate repetitive behavior, as previously described (McFarlane et al., 2008). Briefly, each mouse was placed individually in a clean, empty, cage and given a 10 min habituation period, after which the mice were filmed for another 10 min. Cumulative time spent grooming and number of grooming bouts were counted by an experimenter blinded to the genotypes of the mice.

### Statistical analyses

Data are presented as mean±s.e.m. Statistical analyses were performed using SigmaStat 3.5 software. Student's *t*-test was used to compare two experimental groups and for comparison of several experimental groups, two-way ANOVA or two-way repeated-measures ANOVA were used as required. Tukey's post hoc comparison was used when required.
